# Supplementary material for: Combined laser and ozone therapy for onychomycosis in an in vitro and ex vivo model
Source: PLoS One. 2021 Jun 30;16(6):e0253979. doi: 10.1371/journal.pone.0253979 (PMC8244860; doi:10.1371/journal.pone.0253979)
Supplement: Attachment — Submitted filename: renamed_7d194.pdf [file pone.0253979.s002.pdf]

Departamento de
Biología Funcional
Universidad de Oviedo

ASTURIAS
CAMPUS DE EXCELENCIA
INTERNACIONAL
AD FUTURUM

5th May 2021

Dear Editor,

Please find enclosed the manuscript entitled "COMBINED LASER AND OZONE THERAPY FOR ONYCHOMYCOSIS IN AN *IN VITRO* AND *EX VIVO* MODEL" which is re-submitted to "PLOS ONE" (reference PONE-D-20-35356). This work describes a strong effective novel method for treating onychomycosis infections for the main pathogenic fungi in humans. The developed combined therapy uses two laser wavelengths and ozone gas.

We have accomplished all requirements asked by the referees in the previous revision.

Answers to referees' comments:

Editor's comments:

4. Please ensure that you refer to Figure 2 in your text as, if accepted, production will need this reference to link the reader to the figure.

-Figures 2 and 3 have been substituted by three new figures, which now include the statistical analysis.

Reviewer #1: The manuscript is very well organized, the results are promising; however, minor revision is needed before publication.

1. Please add more up-to-date references in the introduction, and cite them appropriately.

-14 references have been updated.

Campus de El Cristo
Facultad de Medicina y Ciencias de la Salud
C/ Julián Clavería s/n
33006 Oviedo

T. +34 985 10 35 61
F. +34 985 10 35 34
dpto.biofun@uniovi.es

2. I cannot see any statistical analysis in this study, please add the statistical analysis for all of your data

-Statistical analyses have been carried out and 4 new figures with these analyses have been included in the new version (replacing 2 figures and Tables 1 and 2 in the former version).

3. please compare your results with the literature in the discussion

-Our results have been compared with recent papers in the discussion section.

4. The figures are really poor, and are not sharp. I cannot follow the trends in the figure. please upload high quality images.

-Figures quality depends on the PDF conversion generated in the journal webpage during files uploading. We have enclosed high quality TIFF formats in this version, but they will be transformed as well to PDF format.

Minor revision

Reviewer #2: The authors propose to use a combination of 405 nm and 639 nm light for the treatment of onychomycosis in vitro and ex vivo.

The authors need to address my comments before considering publication. Important controls are missing from the study – namely, treatments using light alone. In addition, it would be ideal to evaluate effects of each light wavelength alone (with and without ozone) to understand the role (and potential complementation) of each light wavelength. Also, a serious concern I have is how the fungal inhibition was quantified – the results appear to be qualitative

Campus de El Cristo
Facultad de Medicina y Ciencias de la Salud
C/ Julián Clavería s/n
33006 Oviedo

T. +34 985 10 35 61
F. +34 985 10 35 34
dpto.biofun@uniovi.es

rather than quantitative – thus no statistical significance between each group of tests can be evaluated.

-We have included in this new version, new experiments with treatments using each laser alone and together. Statistical analysis has been carried out in this new version.

Specific comments

Line 92 – please can you add a citation showing NADPH-oxidase as the target for 405 nm light in fungi? What about porphyrins?

-We have included a new reference (Huang et al., 2018) and another one for porphyrins effects (den Hollander et al., 2015).

Line 100 – the use of ozone appears to come out of the blue, I think it would be better to introduce ozone first rather than just say that studies are required to validate efficacy of laser treatment with or without ozone.

-We have changed the position of this paragraph, were ozone is described as antimicrobial, also as onychomycosis treatment.

Line 102 – what treatments? Also please define ppm when first used in text.

-These have been done.

Line 113 – is this the irradiance /cm2? If not, what area? Also, this seems awfully high and I would not classify it as LLLT. Have you measured the thermal effects?

-This is the irradiance per 110 cm2 (the surface area). No thermal effects are detected in the chamber: chamber temperature is always below 30 ºC.

Campus de El Cristo
Facultad de Medicina y Ciencias de la Salud
C/ Julián Clavería s/n
33006 Oviedo

T. +34 985 10 35 61
F. +34 985 10 35 34
dpto.biofun@uniovi.es

Line 115: have you measured transmittance of light through the bag? If so, please include.

-The transmittance has been measured and added in the M&M section.

Line 116: please include the emission spectra

-Data for both emission spectra have been included, showing the minimum, typical and maximum wavelength in each case.

Line 131: on or inside the bag? I assume they should be placed inside. Also, were the lids placed on top? If so, how does the lid and plastic influence transmittance?

-This has been clarified. Inside the bag, without the lids.

Line 133: I would think experiments should be performed at least in triplicate (independently over separate days).

-This has been clarified: triplicates were carried out (not duplicates).

Line 137: why were the control samples incubated for only 48 hours and the treated for 5 days? What is the growth rate of each fungal organism? Would you expect these treatments to attenuate growth rather than inhibit/kill.

-Controls were incubated the same days than the experiments: 7 days. At this time point (7 days) all species (control plates) show good growth rates on the solid media: from 12 mm until 37.5 mm diameter, depending on the fungal species.

Line 139: what was the total radiant exposure of each light wavelength that was exposed? If the irradiance is 1.8 (J/cm2?) this would be a very high radiant exposure 1080-3240 J/cm2. I would be concerned about thermal effects.

-The actual value is 1.8 W/110.36 cm^2, which means 16 mW/cm^2. This has been clarified in the manuscript. No thermal effects are detected in the treatment chamber.

Line 151: was potassium hydroxide not used to dissolve nail keratin and observe the infected nail?

-No, in this work NaOH was not used in the *ex vivo* model.

Line 170: table 1A and B: this method of 'quantification' is not easy for the reader to interpret. What does + vs. ++ mean? We know that +++ means indistinguishable from the control but it is not very helpful. I wonder why did the authors not spread the conidia onto the plate to achieve single colonies so that the CFU might be quantified? To me, this seems far too subjective. In addition, How did the authors determine percent inhibition without quantification?

-New experiments have been carried out during this time, and Tables 1 and 2 have been replaced for the new 4 figures, which include the statistical analyses.

Line 184: I am struggling with the fact that the authors only used the combination of 405 nm and 639 nm under in vitro conditions. For an appropriately designed experiment, they should have also evaluated each respective light wavelength alone to better understand the contribution of each wavelength to the inhibition. The role of the 639 nm wavelength (as per the authors suggestion) is for immunomodulation purposes and to increase blood circulation. In vitro or ex vivo, this is not going to occur. However, it is feasible that 639 nm light might influence the fungal organisms themselves to increase the susceptibility to 405 nm light. Some discussion is needed.

-New experiments have been carried out now, with each of the two lasers independently or together. The statistical analyses of these experiments is included in the new version of the manuscript.

Line 186: why were only 40 ppm and 60 ppm selected. It seemed from the ozone only results, they were very effective alone. it seems only A. terreus was tolerant to this. Ideally, some controls evaluating the role of each wavelength alone should be included. In addition, it seems like light alone (without ozone) are missing?

-The reason is that 20 ppm was poorly effective. 40 and 60 ppm were already quite effective. This allowed to avoid the use of the high dose option (80 ppm), potentially preventing future skin irritations due to the pro-oxidant effect of 80 ppm ozone. Another extra reason is that we wished to detect some potential synergistic effect after including lasers in the ozone treatment. The objective was to select the shorter and less potent treatment option.

Line 214: what is known about immune cell trafficking into the keratin?

-Blood circulation is enhanced in the subungual bed.

line 240: do the authors hypothesize that light is an adjuvant for ozone or ozone is an adjuvant for light?

-Our new results indicate that light alone is inactive, but it enhances the ozone effect in the combined treatments.

Campus de El Cristo
Facultad de Medicina y Ciencias de la Salud
C/ Julián Clavería s/n
33006 Oviedo

T. +34 985 10 35 61
F. +34 985 10 35 34
dpto.biofun@uniovi.es

Departamento de
Biología Funcional
Universidad de Oviedo

ASTURIAS
CAMPUS DE EXCELENCIA
INTERNACIONAL
AD FUTURUM

All co-authors have read the report and are in agreement with its content. None of this work has been submitted or published elsewhere.

Competing Interest

The authors have read the journal's policy and have the following competing interests: IVF is paid employee of Termosalud SL. There are no patents, products in development or marketed products associated with this research to declare. This does not alter our adherence to PLOS ONE policies on sharing data and materials. All other authors declare that they have no competing interests.

Financial Disclosure

This study was supported by the Programa Ayudas a Empresas para la Ejecución de Proyectos de I+D+i en el Principado de Asturias en el Periodo 2014-2015 (IE-14-084) and the Programa de Ayudas a Grupos de Investigación del Principado de Asturias (IDI/2018/000120) in the form of grants to FL. IVF received salary from Termosalud SL. The specific roles of these authors are articulated in the 'author contributions' section. The funders had no role in the study design, data collection and analysis, decision to publish, or preparation of the manuscript.

We thank you for your consideration of this work and we look forward to receiving the new evaluations of this paper.

Sincerely,

Felipe Lombó PhD.
Associate Professor of Microbiology

Prof. Dr. Felipe Lombó Brugos
Área de Microbiología
Departamento de Biología Funcional
Universidad de Oviedo

Campus de El Cristo
Facultad de Medicina y Ciencias de la Salud
C/ Julián Clavería s/n
33006 Oviedo

T. +34 985 10 35 61
F. +34 985 10 35 34
dpto.biofun@uniovi.es

University of Oviedo, SPAIN
email: lombofelipe@uniovi.es
